# Selenium nutritional status and thyroid dysfunction

**DOI:** 10.20945/2359-4292-2023-0348

**Published:** 2025-02-11

**Authors:** Luciana Sant’Ana Leone de Souza, Renata de Oliveira Campos, Jair de Souza Braga Filho, Joice dos Santos de Jesus, Sara Moreira Anunciação, Jéssica Fernanda Cassemiro, Pedro Resende Ferreira Rende, Fábio Hecht, Helton Estrela Ramos

**Affiliations:** 1 Departamento de Biorregulação, Instituto de Saúde e Ciências, Universidade Federal da Bahia, Salvador, BA, Brasil; 2 Programa de Pós-graduação em Processos Interativos de Órgãos e Sistemas, Instituto de Ciências e Saúde, Universidade Federal da Bahia, Salvador, BA, Brasil; 3 Centro de Ciências e Saúde, Recôncavo da Universidade Federal da Bahia, Santo Antonio de Jesus, BA, Brasil; 4 Instituto de Biofísica Carlos Chagas Filho, Universidade Federal do Rio de Janeiro, Rio de Janeiro, RJ, Brasil; 5 Programa de Pós-graduação em Medicina e Saúde, Faculdade de Medicina, Universidade Federal da Bahia, Salvador, BA, Brasil

**Keywords:** Selenium, thyroid, selenium deficiency

## Abstract

Selenium(Se) is an essential micronutrient for several immune and regulatory functions in the body. In thyroid tissue, Se contributes to the antioxidant system and is a crucial component of deiodinases, which are selenoproteins that participate in thyroid hormone metabolism. Additionally, this micronutrient exerts a significant impact on thyroid pathophysiology, as low levels of Se lead to reduced activity of glutathione peroxidase, a selenoprotein involved in antioxidative processes, thereby resulting in increased oxidative stress and damage to thyroid tissue. Selenium deficiency (SeD) can cause growth retardation and reproductive failure; in women and children, it may result in Keshan’s disease and Kashin-Beck’s disease. Research has shown an inverse correlation between Se serum levels and autoimmune thyroiditis in areas with mild SeD. In Graves’ disease, Se supplementation has been linked to faster achievement of euthyroidism as well as improvements in quality of life, lessened orbital involvement, and slower ocular progression of the disease. Furthermore, several studies suggest an association between serum SeD and the development of thyroid cancer. Maintaining physiological Se concentrations appears to be related to the prevention of thyroid disease, although current data are insufficient to conclusively support or refute the efficacy of supplementation. Through this narrative review, we aim to present the latest information on the role of selenium in thyroid pathophysiology. To identify relevant literature, specific search strategies were employed in the electronic databases PubMed, Lilacs, and SciELO.

## INTRODUCTION

Selenium (Se) plays a crucial role in numerous immune and regulatory functions within the human body, including the inactivation of heavy metals, protection against xenobiotics and organic carcinogens, immunomodulation, and the metabolism of prostaglandins, prostacyclin, and thromboxane. Furthermore, Se is vital for the proper functioning of selenoproteins, which are integral to the antioxidant immune system and play a role in preventing chronic diseases (^1^).

The human body can accumulate approximately 10 to 20 mg of Se, with 50% stored in the muscles, kidneys, liver, skeleton, and testes (^2^,^3^). A smaller proportion of Se is found in bones (16%) and blood (10%) (^4^). The thyroid gland, containing the highest Se concentration (0.72 ± 0.44 µg/g), houses several Se-dependent enzymes crucial for hormonal metabolism maintenance, including GPx, TRxs, and iodothyronine deiodinase (DIO) (^3^,^5^). In thyroid tissue, Se fortifies the antioxidant defense system, safeguarding thyroid follicular cells from the excessive hydrogen peroxide (H_2_O_2_) produced during the biosynthesis of THs (^6^).

Selenium also plays a critical role in the functionality of DIO, an enzyme that catalyzes THs, either activating or inactivating them (^7^). Consequently, a deficiency in Se may lead to reduced conversion of thyroxine (T4) to triiodothyronine (T3), the active hormone form (^8^). Notably, a moderate to severe Se deficiency (SeD) has been linked to an increased prevalence of thyroid diseases, including cancer, autoimmune disorders, and nodules (^9^,^10^). This review aims to explore the practical aspects of Se’s impact on the pathophysiology of thyroid diseases, along with dietary sources, nutritional recommendations, and metabolism. To compile relevant literature, specific search strategies were employed in the electronic databases of PubMed, Lilacs, and SciELO.

## FOOD SOURCES AND RECOMMENDED SELENIUM INTAKE

Selenium can be found in both organic compounds, such as selenomethionine (SeMet) and selenocysteine (SeCys), and inorganic forms, such as selenite (SeO_3_^2^) and selenate (SeO_4_^2-^). Sodium selenite (Na_2_SeO_3_), representing SeO_3_^2^, serves as the principal substrate for the hepatic synthesis of selenoproteins (^11^). The concentration of Se in soil significantly influences its presence in food. Additionally, the bioavailability of Se in food is affected by various soil biophysical-chemical parameters, including pH levels and redox potential, Se speciation, texture, mineralogy, microbial activity, organic matter content, and the presence of competing ions (^12^-^14^).

Brazil nuts (*Bertholletia excelsa*, family Lecythidaceae) are exceptionally rich in Se, containing approximately 29.60 µg per gram (^15^). Other foods considered to be good sources of Se include yeast, mushrooms, meat, fish, alfalfa, seafood, liver, kidneys, cereals, and cruciferous vegetables. The bioavailability of Se from these sources varies significantly, ranging from 20% to 50% for seafood and exceeding 80% for cereals and yeast (^16^); SeMet is identified as a predominant component in cereals, yeast, and meat (^17^). Conversely, SeCys is primarily found in foods derived from animals (^18^). Selenium-methylselenocysteine is the principal organic Se compound in vegetables like garlic, onions, stems, and broccoli (^19^). Inorganic Se, present in small quantities in drinking water and various dietary supplements such as sodium selenite, has rates of good bioavailability (^20^,^21^). However, supplements containing SeMet are noted for their superior bioavailability and absorption (^16^,^22^). Table 1 shows the Se concentrations of the main products consumed in Brazil.

**Table 1:** Selenium content in foods consumed in Brazil

Food (100g)	Se (µg)
Brazil nuts	0.03-515
French bread	0.25
Rice	0.04
Eggs (yolk)	0.20
Beef	0.03
Beef liver	7.30
Canned solid tuna	52.50
Canned sardines in oil	46.00
Beans	0.03
Whole milk	0.01
Cheese	0.06
Chicken	0.07
Orange	0.01
Banana	0.01
Minas frescal cheese	9.90
Yogurt	1.70
Brazilian cream cheese	13.00

Source: Phillip Tucunduva Food Composition Table (^12^); Ferreira and cols. (^13^).

The recommended dietary allowance (RDA) for adults is 55 µg/day, a value derived from the Se amount necessary to maximize the synthesis of glutathione peroxidase (GPx). For adults, the maximum tolerable intake level has been established at 800 µg/day, a threshold based on the prevention of adverse effects, including selenosis. The RDA for Se increases to 60 µg/day during pregnancy and to 70 µg/day during lactation (^23^). Table 2 shows the RDA and maximum tolerable levels of Se by age.

**Table 2:** Values for EAR, RDA and UL referring to the selenium intake of children, adolescents and adults

Age (years)	EAR Se (µg/day)	RDA Se (µg/day)	UL Se (µg/day)
1-3	17	20	90
4-8	23	30	150
9-13	35	40	280
14-18	45	55	400
>19	45	55	800

Legend: EAR: Estimated Average Requirement; RDA: Recommended Dietary Allowances; UL: Tolerable Upper Intake Level.

Source: Institute of Medicine (^21^).

## SELENIUM ABSORPTION AND EXCRETION

Organic and inorganic forms of Se are efficiently absorbed by the intestinal epithelium (70%-95%) (^24^). The absorption rate of SeO_4_^2-^ is over 90%, facilitated by a gradient created by Na^+^K^+^ATPase. Notably, a significant portion of SeO_4_^2-^ is excreted through urine before its incorporation into tissues. In contrast, SeO_3_^2-^ exhibits an 80% absorption efficiency in the duodenum through simple diffusion and is better retained in the body than SeO_4_^2-^ (^25^,^26^).

SeMet boasts an absorption efficiency of 95%-98% in the small intestine, facilitated by an active co-transport mechanism involving neutral sodium amino acids. SeCys absorption occurs through an active transport mechanism in conjunction with basic amino acids (^26^). Certain nutrients, such as methionine, vitamins E, A, C, and other antioxidants, are known to enhance the absorption of Se (^26^). Post-absorption, Se compounds are transported to various organs and tissues for selenoprotein synthesis, primarily Selenoprotein P (SePP). The liver and kidneys serve as the central synthesis sites for many selenoproteins, notably SePP and GPx. Subsequently, SePP is released into the bloodstream, playing a critical role in distributing Se from the liver to other organs (^26^). Tissues with high protein synthesis rates, like skeletal muscle, also act as storage sites for Se in the form of SeMet (^20^,^26^).

**Figure 1:**
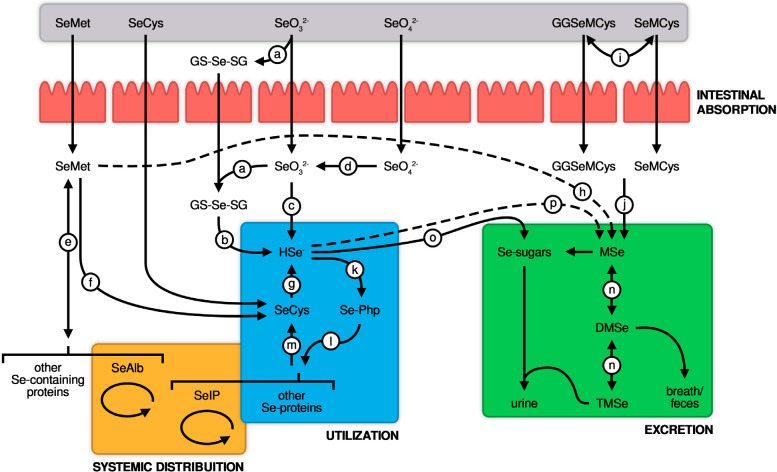
Scheme with global selenium metabolism.

The distribution and bioavailability of Se in tissues are contingent upon its chemical form. Organic and inorganic Se compounds are converted into hydrogen selenite (H_2_Se), which either participates in selenoprotein synthesis or undergoes methylation by thiol S-methyltransferase, leading to the production of methylselenol, dimethyl selenite, and trimethylselenonium (^27^). Conversely, SeMet can be metabolized through different pathways, either meeting the dietary methionine requirements or being converted into SeCys by specific enzymes (^26^,^28^). Global Se metabolism is schematically represented in Figure 1.

## THE PHYSIOLOGICAL FUNCTION OF SELENOPROTEINS

The biological effects of Se are conveyed by at least 30 selenoproteins, each containing a SeCys residue at their catalytic site (^1^). These proteins’ functions have been progressively uncovered and are primarily associated with shielding cells from oxidative harm, facilitating the biosynthesis of deoxynucleoside triphosphate for DNA, and regulating TH metabolism (^8^,^19^,^20^).

The synthesis of selenoproteins is governed by a well-conserved mechanism involving cis-acting elements and trans-acting factors. Se incorporation into proteins follows an unconventional mechanism wherein the UGA stop codon is recoded to function as a sense codon. In certain conditions, particularly in inflammatory diseases, Se levels may decline, leading to compromised synthesis of selenoproteins (^19^). Notably, specific selenoproteins are vital for maintaining thyroid gland homeostasis, as detailed in Table 3.

**Table 3:** Summary of the main selenoproteins that are expressed in the thyroid gland or are involved in hormonal biosynthesis and antioxidant defense and their main function

Selenoproteins	Main function
Glutathione Peroxidase (GPx)	Catalyzes the reduction of H_2_O_2_ and provides protection against oxidative stress
Cytosolic GPx1 (cGPx)	Antioxidant defense
Extracellular GPx (pGPx-3)	Anti-inflammatory action
Phospholipid GPx (GPx-4)	Reduces phospholipid hydroperoxides, moderate apoptosis
Iodothyronine deiodinases (DIs)	Catalyzes the conversion of T_4_ a T_3_ and reverse T_3_
Type 1 deiodinase (D1)	Production of systemic T_3_
Type 2 deiodinase (D2)	Synthesis of intracellular T_3_
Type 3 deiodinase (D3)	Catalyzes the conversion of T_3_ and T_4_ to their inactive derivatives, 3,3' diiodothyronine and rT3
Thyroredoxins reductases (TRx)	Oxidoreductase system with NADPH as a cofactor, modulation of transcription factors and transduction signals
Cytosolic TRx (TRx-1)	Regulates cell proliferation and development
Mitochondrial TRx (TRx-2)	Regulates cell proliferation, tissue development
Selenoprotein P (SePP)	Selenium transport, antioxidant defense
Selenoprotein N (SeP15)	Degradation of H_2_O_2_

Source: Adapted from Papp et al. (^20^) and Drutel et al. (^22^).

Legend: O_2_: Oxygen; H_2_O: Water; H_2_O_2_: hydrogen peroxide; DUOX1: dual oxidase 1; DUOX2: dual oxidase 2; TPO: thyroperoxidase; NOX4: NADPH oxidase 4; GPx: glutathione peroxidase; NRF2: Nuclear factor erythroid 2-related factor 2; SeP15:15-kDa selenoprotein; SePP: selenoprotein P; NIS: sodium/iodide symporter; Trx: thioredoxin; TRXR: thioredoxin reductase; D1: type I deiodinase; D2: type II deiodinase; T3: triiodothyronine; T4: thyroxine; MCT8: monocarboxylate transporter 8.

### The role of glutathione peroxidases

Human GPx constitute a closely related family of antioxidant enzymes, encoded by genes ranging from GP×1 to GP×6 (^29^). GPx1, the most prevalent and initially identified enzyme (^30^,^31^), resides in the cytosol where it acts as an antioxidant by reducing H_2_O_2_ and free organic hydroperoxides into alcohol and water (^32^). Notably, GPx is effective at metabolizing very low H_2_O_2_ concentrations (100 µmol/L), while catalase activity is required for concentrations on the order of mmol/L (^11^).

In the thyroid gland, other expressed glutathione peroxidases include GPx3 and GPx4. GPx3 functions extracellularly, whereas GPx4, also known as phospholipid hydroperoxidase, targets phospholipids and cholesterol hydroperoxides. Moreover, it plays roles in cell shape modulation and apoptosis (^33^-^35^).

Iodine deficiency exacerbates oxidative damage to thyroid tissue, which results in heightened thyroid stimulating hormone (TSH) stimulation and excessive H_2_O_2_ production in thyrocytes amid reduced iodide availability for oxidation (^1^). It is critical to highlight that the expression of these selenoproteins is contingent on Se intake, with studies showing GPX3 activity directly correlating with plasma Se concentration within the 8 to 80 ng Se/mL range (^36^,^37^).

### The role of thioredoxin reductases

There are three main Thioredoxin Reductases: TR1, TR2, and TR3. The principal roles of TRs include modulating redox signaling and antioxidant actions (^38^). The Thioredoxin system is integral to gene expression regulation through the redox modulation of transcription factors such as P-53, NF-kB, Ref-1, AP-1, glucocorticoid receptor, and apoptosis regulating kinase, thus indirectly influencing cellular processes such as proliferation, death, and immunity activation (^39^,^40^). Additionally, TRs can reduce ascorbyl free radicals via ascorbate recycling. Given humans cannot synthesize ascorbic acid, an essential cell-protective antioxidant against oxidative stress and inflammation (^41^), selenoprotein derivatives such as TR are crucial in oxidative stress and inflammation, particularly in wound healing (^42^).

### The role of iodothyronine deiodinases and selenium

Iodothyronine deiodinases are involved in several functions against the background of TH homeostasis. These enzymes, by detaching an iodine atom from the TH molecule, facilitate either its activation or inactivation. In humans, three distinct deiodinases have been identified: iodothyronine deiodinase 1 (D1), D2, and D3. D1 and D2 are primarily associated with the pathway of hormonal activation, whereas D3 engages in the counteractive process of hormonal inactivation (^43^,^44^). The optimal activity of these deiodinases necessitates the presence of Se in the form of SeCys at the catalytic active center. Substitution with Cys markedly diminishes the enzymes’ affinity for their preferred substrate (^45^).

D1, being the most prevalent and thoroughly characterized among the deiodinases, significantly contributes to the generation of active T3 in circulation through the deiodination of T4. It is important to note that while this activity predominantly occurs within the thyroid, it is also present in various other organs, including the liver and kidneys (^44^). In adult mammals, D1 transcripts are identifiable in diverse regions: the pituitary, intestine, placenta, and gonads (^44^,^46^).

D2 facilitates the intracellular production of T3 and is located in the brain, pituitary gland, brown adipose tissue, muscle, and heart. It exhibits a greater affinity for T4 relative to D1, with intracellularly produced T3 being crucial for regulating the hypothalamic-pituitary-thyroid feedback loop (^47^).

D3 serves as the principal physiological inactivator of TH, converting T3 and T4 into their inactive derivatives, 3,3’-diiodothyronine and reverse T3. This enzyme plays a pivotal role in TH homeostasis by guarding tissues against the detrimental effects of excessive active TH (^43^). Its expression is selectively and temporally regulated across different tissues, with a dominant presence in the placenta, central nervous system (CNS), and skin. Within the CNS, D3 aids in maintaining T3 levels. Conversely, in the placenta, it prevents the excessive transplacental transfer of T4 and T3, thereby shielding tissues from premature exposure to TH during embryonic development (^43^).

### Selenoproteins and oxidative system in the biosynthesis of thyroid hormones

The primary steps in hormonal biosynthesis include iodide transport, iodide oxidation, iodide organization, coupling reactions, and T3 and T4 hydrolysis of thyroglobulin (TG) by lysosomal enzymes. Initially, iodide is actively transported from the bloodstream into the thyroid follicle by the sodium-iodide symporter, consuming energy to move against an electrochemical gradient (^47^). Within the cell, iodide is moved across the apical membrane to the follicular lumen by Pendrin and potentially other unidentified systems, in a process termed iodide efflux. Subsequently, iodide is oxidized to iodine by thyroid peroxidase (TPO) at the apical membrane of thyrocytes (^48^).

Hydrogen peroxide is essential as an oxidant in this reaction, catalyzed by TPO. The generation of H_2_O_2_ in thyrocytes is primarily controlled by the availability of iodine and TSH. NADPH oxidase forms the basis of the H_2_O_2_ generating system, with its synthesis at the apical pole of the thyrocytes being catalyzed by dual oxidases 1 and 2 (DUOX1 and DUOX2), utilizing NADPH2 as a coenzyme and stimulated by TSH. This activity is further augmented by the oxidative functions of NADPH oxidase 4 and is inhibited by iodine (^49^).

The intracellular concentration of H_2_O_2_, which is meticulously regulated for signaling purposes, aims to protect against high concentrations in the colloid necessary for iodination of tyrosyl residues in TG to produce monoiodotyrosine and diiodotyrosine residues. Optimal spatial arrangement of DUOX 1 and DUOX 2 complexes, along with superoxide dismutase (SOD), enhances extracellular utilization of H_2_O_2_ and limits its diffusion (^48^). It is hypothesized that excess H_2_O_2_, which can inactivate TPO, is degraded by GPx3 secreted into the colloidal lumen.

Moreover, H_2_O_2_ and other reactive oxygen species (ROS) not fully consumed during TH synthesis are degraded by the antioxidant defensive enzyme system (^30^). Various antioxidant enzymes transform ROS into less harmful compounds, such as cellular GPxs, TRxs, SOD, and catalases. These enzymes collectively form a primary defense against superoxide and H_2_O_2_ (^30^). The subcellular localization of protective selenoproteins within thyrocytes remains to be fully elucidated. However, it is known that extracellular GPx3, or plasma GPx, is one of the predominant selenoproteins in human thyrocytes, contributing significantly to the high Se content in the thyroid. This mechanism seems to directly regulate TH synthesis (^30^). Without TSH, GPx3 secretion at the apical pole of the thyrocyte reduces the amount of H_2_O_2_ available for iodination reactions, thus increasing GPx3 concentrations to enhance protection against oxidative stress induced by TH synthesis (^50^).

All GPxs convert H_2_O_2_ and hydroperoxides to water and oxidized glutathione, using the tripeptide γ-glutamylcysteinylglycine or reduced glutathione (GSH), with one molecule of H_2_O_2_ reduced to two molecules of water in a reaction catalyzed by GPx (^51^). Under normal conditions, most GSH is in its reduced form and is distributed across the nucleus, endoplasmic reticulum, and mitochondria, also acting as a coenzyme for numerous defensive enzymes (^51^). The transcriptional factor NRF2, regulated by the *NFE2L2* gene, is a master regulator of the antioxidant response and controls the expression of countless genes, including those involved in NADPH production, iron sequestration, and the production, utilization, and regeneration of glutathione and thioredoxin, such as Se-containing enzymes thioredoxin reductase 1 and GPX2 (^52^).

**Figure 2:**
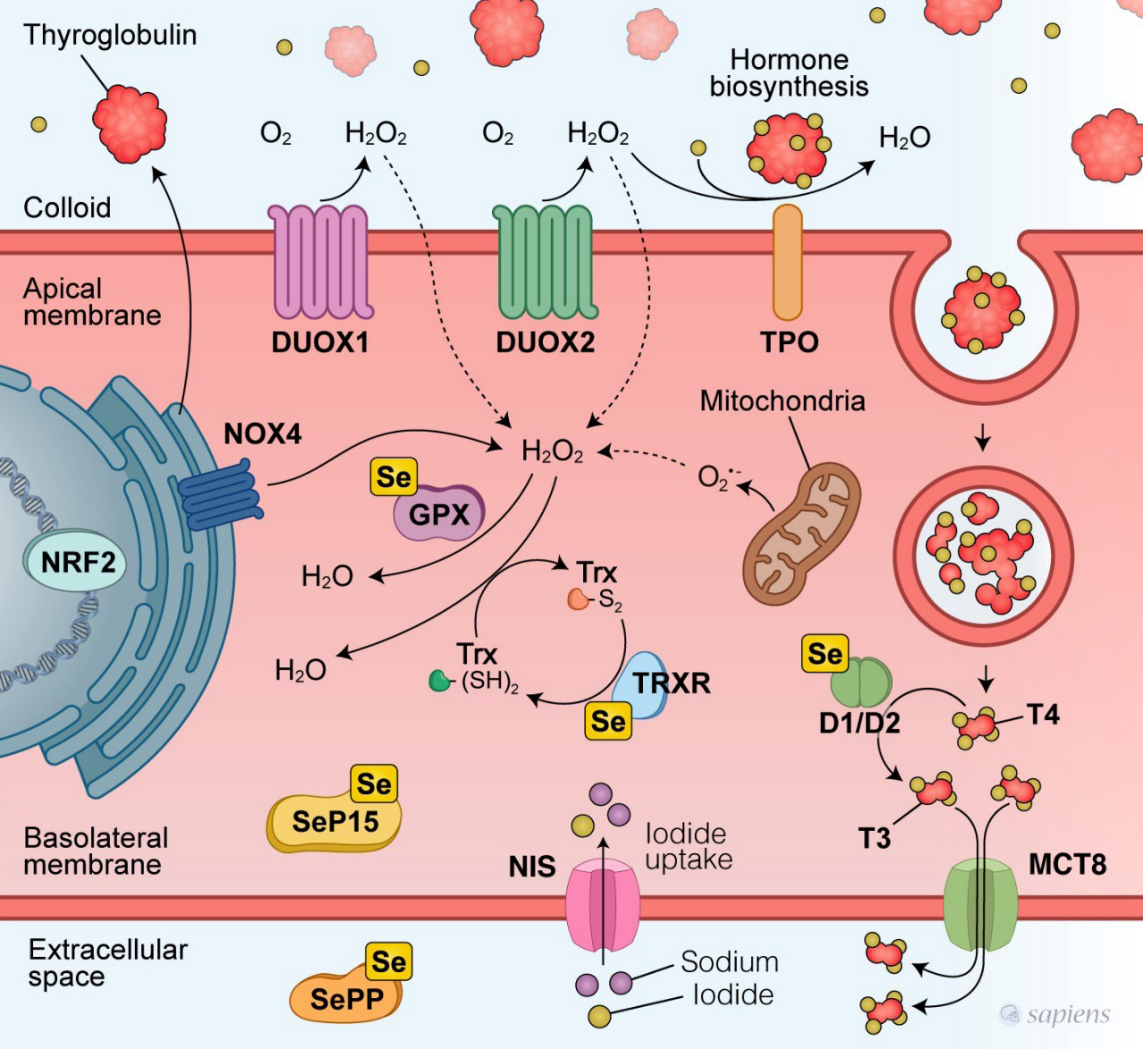
How selenium might contribute to protecting against thyroid dysfunction.

Recent studies have indicated that, within the thyroid gland, NRF2 also positively regulates basal and TSH-induced expression of TG and is essential in protecting the thyroid from iodide overload-induced oxidative stress (^53^). Furthermore, SeD has been shown to activate NRF2 signaling in certain animal models (^54^); however, the effects of SeD on NRF2 signaling in patients unclear. Figure 2 shows the main components of the oxidative system in thyrocytes and the selenoproteins responsible for antioxidant defense.

## SELENIUM DEFICIENCY

Selenium deficiency becomes evident when intake is less than 30 µg/day. Two diseases associated with SeD, Keshan’s disease and Kashin-Beck’s disease, have been documented. Keshan’s disease, prevalent in certain regions of China, manifests as juvenile cardiomyopathy related to severely low Se intake (<15 µg/day), predominantly affecting fertile women and children aged 2-7 years (^50^). Kashin-Beck’s disease is a chronic osteoarthropathy leading to joint deformities. While its etiology remains unclear, studies have associated environmental SeD with the condition, impacting children aged 3-12 years, regardless of sex (^55^-^58^). The specific duration of low Se intake required to influence the development of these diseases, however, has yet to be determined.

## SELENIUM TOXICITY

Excessive Se consumption is observed in locations with a high content of this mineral in the soil. Some of the clinical manifestations of intoxication are asthenia, nausea, diarrhea, alopecia, fragile nails, infertility, and garlic-like odor breath. Neurological disorders and chronic Se supplementation can lead to selenosis, causing liver damage (^59^). Chronic exposure to high Se levels has been associated with an increased mortality risk (^60^), and acute Se poisoning (1-100 mg Se/kg) has been linked to deaths (^61^).

The precise duration of high Se intake necessary to affect disease development remains unknown. Despite inconclusive clinical data on adverse effects, Se intoxication negatively impacts endocrine system functions, including impaired synthesis of TH, growth hormone, sex steroid hormones, insulin-like growth factor 1, and elevating type 2 diabetes mellitus risk (^54^). Regional Se levels for protection against selenosis are 400-480 µg/L in whole blood, 180-230 µg/L in plasma, and 90-110 µg/L in urine. Selenosis risk is linked to micronutrient intakes exceeding 400 mg/day (^62^).

## SELENIUM AND THYROID DISEASES

### Thyroid autoimmunity, hypothyroidism, and selenium

The etiopathogenesis of autoimmune thyroiditis is multifaceted, with evidence suggesting that in individuals genetically predisposed, the onset of autoimmune inflammatory pathogenesis in the thyroid gland may be associated with excessive iodine intake, coupled with Se and iron deficiencies, certain drugs affecting the immune system, and viral infections (^9^). Excessive iodine can impair thyroid function or elevate the risk of thyroiditis in individuals with existing autoimmunity by increasing the production of ROS while diminishing internal antioxidant levels (^63^,^64^).

Nonetheless, the advantages of iodine prophylaxis in mitigating iodine deficiency disorders in areas with inadequate intake surpass the instances of autoimmune thyroiditis (AT) and hyperthyroidism observed in the general populace (^65^). An inverse relationship has been documented between Se plasma levels and the incidence of autoimmune thyroiditis in areas with mild SeD. Consequently, dietary SeD might trigger and sustain autoimmune thyroiditis in susceptible individuals. Notably, severe SeD in mice is linked with increased intraglandular necrosis and macrophage infiltration, likely due to an intensified inflammatory process in the thyroid resulting from diminished GPx activity and the absence of Se’s immunomodulatory effects (^66^).

Several studies suggest Se supplementation may benefit patients with Hashimoto’s thyroiditis. Remarkably, Nacamulli and cols. observed a reduction in thyroid echogenicity after six months and a decrease in thyroglobulin antibody (TgAb) levels after 12 months in patients with autoimmune thyroiditis receiving 80 µg/day of sodium selenite, with no observed increase in TSH levels, indicating stable thyroid function (^67^). Similarly, Gärtner and cols. found that a 200 µg/day dose of sodium selenite reduced thyroid peroxidase antibody (TPOAb) levels and improved thyroid echogenicity (^68^). Turker and cols. reported more favorable outcomes with doses of 100 and 200 µg/day of SeMet, suggesting that doses greater than 100 µg/day are necessary to enhance GPx activity, although a decreased rate of TPOAb suppression over time was noted (^69^).

Duntas and cols. conducted a randomized, placebo-controlled trial involving 65 patients with AT, where the group supplemented with SeMet exhibited a 46% decrease in TPOAb levels after three months and a 55.5% decrease after six months, in contrast to only a 21% and 27% decrease, respectively, in the group receiving only L-thyroxine therapy (^70^). Conversely, an Italian study found that short-term administration of L-SeMet (166 µg/day orally for six months) had a limited effect on the progression of Hashimoto’s thyroiditis (HT), as indicated by unchanged levels of TSH, TPOAb, and CXCL10, a chemokine crucial to the immune pathogenesis of HT (^71^).

Kvicala and cols. also demonstrated that supplementation with Se-rich yeast extract did not reduce TPOAb concentrations (^72^), and sodium selenite supplementation in Dutch patients had no impact on TPOAb levels (^73^). Ferrari and cols. observed an immunomodulatory effect of myo-inositol in conjunction with SeMet in patients with AT, resulting in a significant decline in TSH levels in the supplemented group (^74^). A 2014 Cochrane systematic review summary highlighted that Se supplementation effectively reduces serum TPOAb levels at 3, 6, and 12 months, and serum TgAb levels at 12 months in populations treated with levothyroxine (LT4). However, no significant correlation was found between baseline serum Se levels and the decrease in serum TPOAb levels in LT4-treated patients. This meta-analysis also revealed that the reduction in serum TPOAb levels was only observed in patients receiving 200 µg of SeMet, not in those receiving 200 µg of sodium selenite (^6^). Another meta-analysis and systematic review concluded that current data is insufficient to definitively support or oppose the efficacy of Se supplementation in patients with AT (^75^-^77^).

### Subclinical hypothyroidism

Andrade and cols. (^78^) evaluated the association between dietary Se intake and subclinical hypothyroidism (SCH). This evaluation was based on the database of the Longitudinal Study of Adult Health in Brazil (ELSA-Brazil), encompassing a final sample of 14,283 employees of both sexes, aged between 35 and 74 years. The prevalence of SCH in the studied sample was 5.4%, and a negative correlation was identified between Se consumption and SCH (^78^).

Pirola and cols. conducted the SETI study, a prospective investigation assessing the impact of Se supplementation on TSH and interferon-inducible chemokines (CXCL9, CXCL10, and CXCL11) levels in patients with SCH due to Hashimoto’s thyroiditis. In the group supplemented with 83 mcg of SeMet per day for four months, euthyroidism was restored in 48.9% of participants (22/45), while 23 patients remained hypothyroid. No significant changes were observed in TPOAb, CXCL9, CXCL10, and CXCL11 levels from baseline to the study’s conclusion in both groups. Six months after discontinuing SeMet, 83.3% of the respondents maintained euthyroid status, whereas only 14.2% of the non-responders achieved euthyroidism (^79^). Moreover, administering an oral dose of 83 µg/day of SeMet for four months resulted in significantly more participants restoring euthyroidism in the treatment group compared to controls (31.3% *vs.* 3.1%, p < 0.0001) (^80^).

Payer and cols. demonstrated in a recent study that patients with SCH experienced significant improvements in their condition upon receiving a combination of myo-inositol and Se for six months. Improvements were noted in TSH values, autoimmunity indices, and thyroid status. Additionally, significant enhancements in symptom perception associated with SCH were observed throughout the treatment period (^81^).

Conversely, a double-blinded, randomized, placebo-controlled clinical trial involving 42 patients revealed that Se supplementation with 200 µg of Se for eight weeks did not significantly influence serum TPOAb and TSH levels in patients with SCH (^82^).

The relationship between hypothyroidism and oxidative stress might stem from the decreased activity of the internal antioxidant system, which fails to protect cells from the accumulation of free radicals, resulting in oxidative damage. This accumulation can interfere with TPO activity, disrupting TH production and leading to hypothyroidism. Also, mutations in *DUOX1* or *DUOX2* genes can induce hypothyroidism due to inadequate H_2_O_2_ production (^63^,^64^).

### Graves’ disease

Graves’ disease (GD) is an autoimmune disorder characterized by hyperthyroidism and involves the binding of anti-TSH receptor antibodies to the TSH receptor, stimulating excessive TH production and thyroid hypertrophy. The primary treatment approach for GD is antithyroid drugs, which present a high recurrence rate (^83^). The disease is associated with an increased basal metabolic state and consequent elevation in free radical production, highlighting an imbalance between oxidizing and antioxidant agents. This imbalance underscores oxidative stress as a contributing factor to autoimmunity (^84^).

Selenoproteins, including GPx, play a pivotal role in thyroid autoimmunity, with SeD significantly impacting the initiation and progression of autoimmune thyroid diseases (^85^). GPx, a selenoprotein containing SeCys in its structure, acts as a catalyst in reducing H_2_O_2_ and lipid hydroperoxide, emphasizing Se’s importance in managing oxidative stress and related proinflammatory cytokines (^86^).

Serum Se measurements have been linked to the recurrence or development of orbitopathy, with increased levels observed in individuals achieving remission and reduced levels in those with hyperthyroidism (^87^-^90^). Supplementation with Se alongside methimazole has indicated quicker attainment of euthyroidism in GD patients with moderate SeD compared to methimazole treatment alone (^84^). However, Leo and cols. reported no effect of 166 g/day adjuvant SeMet for three months in 30 GD patients (^91^).

Additionally, Se therapy has shown a beneficial effect on thyroid eye disease (TED) (^87^). A randomized, double-blind study involving 159 patients with mild TED demonstrated that sodium selenite administration (100 µg, twice daily, for six months) led to an improvement in quality of life, disease progression reduction, and decreased ocular involvement compared to placebo and pentoxifylline groups. Remarkably, these effects persisted six months post-supplementation discontinuation (^85^). Following this study, the European Group of Graves’ Orbitopathy guidelines recommended Se supplementation in mild TED cases (^92^). Nevertheless, the severity and activity of TED seem unaffected by Seor selenoprotein P concentrations (^93^).

### Nodular goiter

Although the cause remains unknown, studies suggest that SeD may be a possible risk factor for the development of thyroid nodules. However, determining the role of Se in the etiology of nodular goiter is challenging in clinical research due to significant variations in Se concentrations across different populations (^94^). Keshteli and cols. assessed the role of SeD in the etiology of goiter in children from Isfahan, Iran, finding that plasma Se levels were notably lower in those diagnosed with goiter compared to children without it (^94^).

Rasmussen and cols. demonstrated that a low serum Se concentration tended to increase the risk for multiple nodules larger than 10 mm in diameter (p = 0.087), while serum Se levels did not influence the risk for solitary nodules (p = 0.855) (^5^). Additionally, a study conducted in France involving 792 men and 1,108 women observed an inverse relationship between Se status and thyroid volume in women (p = 0.003), as well as a protective effect of Se against goiter and damage to thyroid tissue (^95^). In Danish adults, low serum Se was significantly associated with an increased tendency to develop multiple nodules (^96^).

Nonetheless, Sakız and cols. investigated, in an iodine sufficient area, the relationship between Se levels, multinodular goiter (70 patients), solitary nodules (70 patients), and patients without nodules (60 patients). The mean serum Se level of all patients included in the study was 57.9 ± 14.4 µg/L, and no significant relationship was observed between serum Se levels and nodular thyroid disease (^97^).

Similarly, a Chinese study aimed to investigate the prevalence of thyroid disease in areas with different Se soil concentrations showed no significant difference in the prevalence of thyroid nodules, yet a higher prevalence of subclinical hypothyroidism and autoimmune thyroiditis was observed in areas with lower Se concentration (^14^). Despite these findings, further research is necessary to establish a definitive correlation between nodular goiter and Se levels.

### Thyroid cancer

Although the mechanisms are not fully understood, Se appears to exhibit anti-carcinogenic activities (^98^), specifically through the action of GPx and thioredoxin reductases (TrxR), which are important for reducing the generation of ROS and protecting DNA and cellular components from free radicals. Selenium is also believed to stimulate the activation of tumor suppressor protein p53, inhibiting cell proliferation, promoting apoptosis, and facilitating DNA repair (^99^). Oxidative stress in thyroid cancer is higher than in healthy tissues, and a decrease in GPx1 and TrxR1 in carcinoma indicates an inadequate antioxidant system response to free radicals (^100^).

Similarly, Se levels are found in smaller amounts in thyroid cancers compared to benign conditions (^101^,^102^), yet establishing a cause-effect relationship between Se and thyroid cancer remains inconclusive (^103^-^106^). In a meta-analysis by Shen and cols., which included 1,291 patients, lower serum Se concentration was observed in individuals with thyroid carcinoma, suggesting that SeD may be a risk factor for thyroid cancer (^101^). Conversely, another study reported an association between the advanced stage of the disease and lower Se concentrations when evaluating 65 patients (^102^). Nonetheless, systematic reviews, including one by de Oliveira Maia and cols. that encompassed five cross-sectional studies (^106^), indicated varying results regarding serum Se concentration and thyroid cancer, highlighting the need for additional research to understand the association between Se levels and thyroid cancer pathophysiology.

In conclusion, maintaining physiological selenium concentration is associated with the prevention of thyroid disease and general health. Selenium supplementation in cases of deficiency may benefit the immune mechanisms of patients with autoimmune thyroiditis or subclinical hypothyroidism, though evidence to support or refute the effectiveness of supplementation is still inadequate. Selenium supplementation has shown benefits in patients with mild to moderate thyroid eye disease.

## References

[1] Winther KH, Rayman MP, Bonnema SJ, Hegedüs L. (2020). Selenium in thyroid disorders - essential knowledge for
clinicians. Nat Rev Endocrinol.

[2] Castro WM. (2007). Selenio en los pacientes críticos con respuesta
inflamatoria sistémica. Nutr Hosp.

[3] Tinggi U. (2008). Selenium: its role as antioxidant in human health. Environ Health Prev Med.

[4] Zachara BA, Pawluk H, Bloch-boguslawska E, Śliwka KM, Korenkiewicz J, Skok Ź (2001). Tissue Level, Distribution, and Total Body Selenium Content in
Healthy and Diseased Humans in Poland. Arch Environ Health.

[5] Rasmussen LB, Schomburg L, Köhrle J, Pedersen IB, Hollenbach B, Hög A (2011). Selenium status, thyroid volume, and multiple nodule formation in
an area with mild iodine deficiency. Eur J Endocrinol.

[6] van Zuuren EJ, Albusta AY, Fedorowicz Z, Carter B, Pijl H. (2014). Selenium Supplementation for Hashimoto’s Thyroiditis: Summary of
a Cochrane Systematic Review. Eur Thyroid J.

[7] Schmutzler C, Mentrup B, Schomburg L, Hoang-Vu C, Herzog V, Köhrle J. (2007). Selenoproteins of the thyroid gland: expression, localization and
possible function of glutathione peroxidase 3. Biol Chem.

[8] Schweizer U, Chiu J, Köhrle J. (2008). Peroxides and Peroxide-Degrading Enzymes in the
Thyroid. Antioxid Redox Signal.

[9] Hu S, Rayman MP. (2017). Multiple Nutritional Factors and the Risk of Hashimoto’s
Thyroiditis. Thyroid.

[10] Wang Y, Zhao F, Rijntjes E, Wu L, Wu Q, Sui J (2019). Role of Selenium Intake for Risk and Development of
Hyperthyroidism. J Clin Endocrinol Metab.

[11] Aaseth J, Frey H, Glattre E, Norheim G, Ringstad J, Thomassen Y. (1990). Selenium concentrations in the human thyroid
gland. Biol Trace Elem Res.

[12] Tolu J, Thiry Y, Bueno M, Jolivet C, Potin-Gautier M, Le Hécho I. (2014). Distribution and speciation of ambient selenium in contrasted
soils, from mineral to organic rich. Sci Total Environ.

[13] Fordyce F. (2007). Selenium geochemistry and health. Ambio.

[14] Li Z, Liang D, Peng Q, Cui Z, Huang J, Lin Z. (2017). Interaction between selenium and soil organic matter and its
impact on soil selenium bioavailability: A review. Geoderma.

[15] Philippi ST. (2020). Tabela de composição de alimentos: suporte para
decisão nutricional.

[16] Ventura M, Melo M, Carrilho F. (2017). Selenium and Thyroid Disease: From Pathophysiology to
Treatment. Int J Endocrinol.

[17] Yang R, Liu Y, Zhou Z. (2017). Selenium and Selenoproteins, from Structure, Function to Food
Resource and Nutrition. Food Sci Technol Res.

[18] Weeks BS, Hanna MS, Cooperstein D. (2012). Dietary selenium and selenoprotein function. Med Sci Monit.

[19] Papp LV, Lu J, Holmgren A, Khanna KK. (2007). From Selenium to Selenoproteins: Synthesis, Identity, and Their
Role in Human Health. Antioxid Redox Signal.

[20] Drutel A, Archambeaud F, Caron P. (2013). Selenium and the thyroid gland: more good news for
clinicians. Clin Endocrinol (Oxf).

[21] World Health Organization (1996). Trace elements in human nutrition and health.

[22] Kubachka KM, Hanley T, Mantha M, Wilson RA, Falconer TM, Kassa Z (2017). Evaluation of selenium in dietary supplements using elemental
speciation. Food Chem.

[23] Kieliszek M, Błażejak S. (2013). Selenium: Significance, and outlook for
supplementation. Nutrition.

[24] Nóbrega PT. (2015). Selénio e a importância para o organismo humano:
benefícios e controvérsias [Mestrado].

[25] Dumont E, Vanhaecke F, Cornelis R. (2006). Selenium speciation from food source to metabolites: a critical
review. Anal Bioanal Chem.

[26] Thiry C, Ruttens A, De Temmerman L, Schneider YJ, Pussemier L. (2012). Current knowledge in species-related bioavailability of selenium
in food. Food Chem.

[27] Suzuki KT, Tsuji Y, Ohta Y, Suzuki N. (2008). Preferential organ distribution of methylselenol source
Se-methylselenocysteine relative to methylseleninic acid. Toxicol Appl Pharmacol.

[28] Fairweather-Tait SJ, Bao Y, Broadley MR, Collings R, Ford D, Hesketh JE (2011). Selenium in Human Health and Disease. Antioxid Redox Signal.

[29] Ruseva B, Himcheva I, Nankova D. (2013). Importance of selenoproteins for the function of the thyroid
gland. Sci Technol.

[30] Beckett GJ, Arthur JR. (2005). Selenium and endocrine systems. J Endocrinol.

[31] Reilly C. (2006). Selenium in Food and Health.

[32] Jackson MI, Lunøe K, Gabel-Jensen C, Gammelgaard B, Combs GF. (2013). Metabolism of selenite to selenosugar and trimethylselenonium in
vivo: tissue dependency and requirement for S-adenosylmethionine-dependent
methylation. J Nutr Biochem.

[33] Imai H, Nakagawa Y. (2003). Biological significance of phospholipid hydroperoxide glutathione
peroxidase (PHGPx, GPx4) in mammalian cells. Free Radic Biol Med.

[34] Moghadaszadeh B, Beggs AH. (2006). Selenoproteins and Their Impact on Human Health Through Diverse
Physiological Pathways. Physiology (Bethesda).

[35] Köhrle J, Jakob F, Contempré B, Dumont JE. (2005). Selenium, the Thyroid, and the Endocrine System. Endocr Rev.

[36] Cozzolino SMF. (2012). Biodisponibilidade de Nutrientes.

[37] Clausen J, Nielsen SA. (1988). Selenium.

[38] Ducros V, Favier A. (2004). Métabolisme du sélénium. EMC - Endocrinologie.

[39] Rundlöf AK, Arnér ESJ. (2004). Regulation of the Mammalian Selenoprotein Thioredoxin Reductase 1
in Relation to Cellular Phenotype, Growth, and Signaling
Events. Antioxid Redox Signal.

[40] Selenius M, Rundlöf AK, Olm E, Fernandes AP, Björnstedt M. (2010). Selenium and the Selenoprotein Thioredoxin Reductase in the
Prevention, Treatment and Diagnostics of Cancer. Antioxid Redox Signal.

[41] Mustacich D, Powis G. (2000). Thioredoxin reductase. Biochem J.

[42] Hariharan S, Dharmaraj S. (2020). Selenium and selenoproteins: it’s role in regulation of
inflammation. Inflammopharmacology.

[43] Dentice M, Salvatore D. (2011). Deiodinases: the balance of thyroid hormone: Local impact of
thyroid hormone inactivation. J Endocrinol.

[44] Sabatino L, Vassalle C, Del Seppia C, Iervasi G. (2021). Deiodinases and the Three Types of Thyroid Hormone Deiodination
Reactions. Endocrinol Metab (Seoul).

[45] Bianco AC, Salvatore D, Gereben B, Berry MJ, Larsen PR. (2002). Biochemistry, Cellular and Molecular Biology, and Physiological
Roles of the Iodothyronine Selenodeiodinases. Endocr Rev.

[46] Maia AL, Goemann IM, Meyer ELS, Wajner SM. (2011). Deiodinases: the balance of thyroid hormone: Type 1 iodothyronine
deiodinase in human physiology and disease. J Endocrinol.

[47] Arrojo e Drigo R, Bianco AC. (2011). Type 2 deiodinase at the crossroads of thyroid hormone
action. Int J Biochem Cell Biol.

[48] Schomburg L. (2011). Selenium, selenoproteins and the thyroid gland: interactions in
health and disease. Nat Rev Endocrinol.

[49] Massart C, Hoste C, Virion A, Ruf J, Dumont JE, Van Sande J. (2011). Cell biology of H2O2 generation in the thyroid: Investigation of
the control of dual oxidases (DUOX) activity in intact ex vivo thyroid
tissue and cell lines. Mol Cell Endocrinol.

[50] Köhrle J, Jakob F, Contempré B, Dumont JE. (2005). Selenium, the Thyroid, and the Endocrine System. Endocr Rev.

[51] Quintana-Cabrera R, Bolaños JP. (2013). Glutathione and γ-Glutamylcysteine in Hydrogen Peroxide
Detoxification. Methods Enzymol.

[52] Taguchi K, Motohashi H, Yamamoto M. (2011). Molecular mechanisms of the Keap1-Nrf2 pathway in stress response
and cancer evolution. Genes Cells.

[53] Ziros PG, Habeos IG, Chartoumpekis DV, Ntalampyra E, Somm E, Renaud CO (2018). NFE2-Related Transcription Factor 2 Coordinates Antioxidant
Defense with Thyroglobulin Production and Iodination in the Thyroid
Gland. Thyroid.

[54] Brigelius-Flohé R, Kipp AP. (2013). Selenium in the Redox Regulation of the Nrf2 and the Wnt
Pathway. Methods Enzymol.

[55] Liu H, Yu F, Shao W, Ding D, Yu Z, Chen F (2018). Associations Between Selenium Content in Hair and Kashin-Beck
Disease/Keshan Disease in Children in Northwestern China: a Prospective
Cohort Study. Biol Trace Elem Res.

[56] Rayman MP. (2020). Selenium intake, status, and health: a complex
relationship. Hormones (Athens).

[57] Campa A, Baum M, Bussmann H, Martinez S, Farahani M, van Widenfelt E (2017). The effect of micronutrient supplementation on active TB
incidence early in HIV infection in Botswana. Nutr Diet Suppl.

[58] Baum MK, Campa A, Lai S, Sales Martinez S, Tsalaile L, Burns P (2013). Effect of Micronutrient Supplementation on Disease Progression in
Asymptomatic, Antiretroviral-Naive, HIV-Infected Adults in
Botswana. JAMA.

[59] Triggiani V, Tafaro E, Giagulli V, Sabba C, Resta F, Licchelli B (2009). Role of Iodine, Selenium and Other Micronutrients in Thyroid
Function and Disorders. Endocr Metab Immune Disord Drug Targets.

[60] Vinceti M, Ballotari P, Steinmaus C, Malagoli C, Luberto F, Malavolti M (2016). Long-term mortality patterns in a residential cohort exposed to
inorganic selenium in drinking water. Environ Res.

[61] Hadrup N, Ravn-Haren G. (2020). Acute human toxicity and mortality after selenium ingestion: A
review. J Trace Elem Med Biol.

[62] Institute of Medicine (US) Panel on Dietary Antioxidants and Related
Compounds (2000). Dietary Reference Intakes for Vitamin C, Vitamin E, Selenium, and
Carotenoids.

[63] Kochman J, Jakubczyk K, Bargiel P, Janda-Milczarek K. (2021). The Influence of Oxidative Stress on Thyroid
Diseases. Antioxidants (Basel).

[64] Ohye H, Sugawara M. (2010). Dual oxidase, hydrogen peroxide and thyroid
diseases. Exp Biol Med (Maywood).

[65] Rayman MP. (2019). Multiple nutritional factors and thyroid disease, with particular
reference to autoimmune thyroid disease. Proc Nutr Soc.

[66] Pearce EN, Farwell AP, Braverman LE. (2003). Thyroiditis. N Engl J Med.

[67] Nacamulli D, Mian C, Petricca D, Lazzarotto F, Barollo S, Pozza D (2010). Influence of physiological dietary selenium supplementation on
the natural course of autoimmune thyroiditis. Clin Endocrinol (Oxf).

[68] Gärtner R, Gasnier BCH, Dietrich JW, Krebs B, Angstwurm MWA. (2002). Selenium Supplementation in Patients with Autoimmune Thyroiditis
Decreases Thyroid Peroxidase Antibodies Concentrations. J Clin Endocrinol Metab.

[69] Turker O, Kumanlioglu K, Karapolat I, Dogan I. (2006). Selenium treatment in autoimmune thyroiditis: 9-month follow-up
with variable doses. J Endocrinol.

[70] Duntas L, Mantzou E, Koutras D. (2003). Effects of a six month treatment with selenomethionine in
patients with autoimmune thyroiditis. Eur J Endocrinol.

[71] Esposito D, Rotondi M, Accardo G, Vallone G, Conzo G, Docimo G (2017). Influence of short-term selenium supplementation on the natural
course of Hashimoto’s thyroiditis: clinical results of a blinded
placebo-controlled randomized prospective trial. J Endocrinol Invest.

[72] Kvicala J, Hrdá P, Zamrazil V, Nemecek J, Hill M, Jiranek V. (2009). Effect of selenium supplementation on thyroid
antibodies. J Radioanal Nucl Chem.

[73] Eskes SA, Endert E, Fliers E, Birnie E, Hollenbach B, Schomburg L (2014). Selenite supplementation in euthyroid subjects with thyroid
peroxidase antibodies. Clin Endocrinol (Oxf).

[74] Ferrari SM, Fallahi P, Di Bari F, Vita R, Benvenga S, Antonelli A. (2017). Myo-inositol and selenium reduce the risk of developing overt
hypothyroidism in patients with autoimmune thyroiditis. Eur Rev Med Pharmacol Sci.

[75] Qiu Y, Xing Z, Xiang Q, Yang Q, Zhu J, Su A. (2021). Insufficient evidence to support the clinical efficacy of
selenium supplementation for patients with chronic autoimmune
thyroiditis. Endocrine.

[76] Winther KH, Wichman JEM, Bonnema SJ, Hegedüs L. (2017). Insufficient documentation for clinical efficacy of selenium
supplementation in chronic autoimmune thyroiditis, based on a systematic
review and meta-analysis. Endocrine.

[77] Wichman J, Winther KH, Bonnema SJ, Hegedüs L. (2016). Selenium Supplementation Significantly Reduces Thyroid
Autoantibody Levels in Patients with Chronic Autoimmune Thyroiditis: A
Systematic Review and Meta-Analysis. Thyroid.

[78] Andrade G, Gorgulho B, Lotufo P, Bensenor I, Marchioni D. (2018). Dietary Selenium Intake and Subclinical Hypothyroidism: A
Cross-Sectional Analysis of the ELSA-Brasil Study. Nutrients.

[79] Pirola I, Rotondi M, Cristiano A, Maffezzoni F, Pasquali D, Marini F (2020). Selenium supplementation in patients with subclinical
hypothyroidism affected by autoimmune thyroiditis: Results of the SETI
study. Endocrinol Diabetes Nutr (Engl Ed).

[80] Pirola I, Gandossi E, Agosti B, Delbarba A, Cappelli C. (2016). Selenium supplementation could restore euthyroidism in
subclinical hypothyroid patients with autoimmune thyroiditis. Endokrynol Pol.

[81] Payer J, Jackuliak P, Kužma M, Džupon M, Vaňuga P. (2022). Supplementation with myo-inositol and Selenium improves the
clinical conditions and biochemical features of women with or at risk for
subclinical hypothyroidism. Front Endocrinol (Lausanne).

[82] Mahmoudi L, Mobasseri M, Ostadrahimi A, Pourmoradian S, Soleimanzadeh H, Kafili B. (2021). Effect of selenium-enriched yeast supplementation on serum
thyroid-stimulating hormone and anti-thyroid peroxidase antibody levels in
subclinical hypothyroidism: Randomized controlled trial. Adv Biomed Res.

[83] Wang L, Wang B, Chen S, Hou X, Wang X, Zhao S (2016). Effect of Selenium Supplementation on Recurrent Hyperthyroidism
Caused by Graves’ Disease: A Prospective Pilot Study. Horm Metab Res.

[84] Vrca VB, Skreb F, Cepelak I, Romic Z, Mayer L. (2004). Supplementation with antioxidants in the treatment of Graves’
disease; the effect on glutathione peroxidase activity and concentration of
selenium. Clin Chim Acta.

[85] Marinò M, Menconi F, Rotondo Dottore G, Leo M, Marcocci C. (2018). Selenium in Graves Hyperthyroidism and
Orbitopathy. Ophthalmic Plast Reconstr Surg.

[86] Ye R, Huang J, Wang Z, Chen Y, Dong Y. (2022). The Role and Mechanism of Essential Selenoproteins for
Homeostasis. Antioxidants (Basel).

[87] Marcocci C, Kahaly GJ, Krassas GE, Bartalena L, Prummel M, Stahl M (2011). Selenium and the Course of Mild Graves’
Orbitopathy. N Engl J Med.

[88] Wertenbruch T, Willenberg HS, Sagert C, Nguyen TB, Bahlo M, Feldkamp J (2007). Serum selenium levels in patients with remission and relapse of
graves’ disease. Med Chem.

[89] Khong JJ, Goldstein RF, Sanders KM, Schneider H, Pope J, Burdon KP (2014). Serum selenium status in Graves’ disease with and without
orbitopathy: a case-control study. Clin Endocrinol (Oxf).

[90] Bülow Pedersen I, Knudsen N, Carlé A, Schomburg L, Köhrle J, Jørgensen T (2013). Serum selenium is low in newly diagnosed Graves’ disease: a
population-based study. Clin Endocrinol (Oxf).

[91] Leo M, Bartalena L, Rotondo Dottore G, Piantanida E, Premoli P, Ionni I (2017). Effects of selenium on short-term control of hyperthyroidism due
to Graves’ disease treated with methimazole: results of a randomized
clinical trial. J Endocrinol Invest.

[92] Bartalena L, Kahaly GJ, Baldeschi L, Dayan CM, Eckstein A, Marcocci C (2021). The 2021 European Group on Graves’ orbitopathy (EUGOGO) clinical
practice guidelines for the medical management of Graves’
orbitopathy. Eur J Endocrinol.

[93] Dehina N, Hofmann PJ, Behrends T, Eckstein A, Schomburg L. (2016). Lack of Association between Selenium Status and Disease Severity
and Activity in Patients with Graves’ Ophthalmopathy. Eur Thyroid J.

[94] Keshteli AH, Hashemipour M, Siavash M, Amini M. (2009). Selenium Deficiency as a Possible Contributor of Goiter in
Schoolchildren of Isfahan, Iran. Biol Trace Elem Res.

[95] Derumeaux H, Valeix P, Castetbon K, Bensimon M, Boutron-Ruault M, Arnaud J (2003). Association of selenium with thyroid volume and echostructure in
35- to 60-year-old French adults. Eur J Endocrinol.

[96] Rasmussen LB, Hollenbach B, Laurberg P, Carlé A, Hög A, Jørgensen T (2009). Serum selenium and selenoprotein P status in adult Danes - 8-year
followup. J Trace Elem Med Biol.

[97] Sakız D, Kaya A, Kulaksizoglu M. (2016). Serum Selenium Levels in Euthyroid Nodular Thyroid
Diseases. Biol Trace Elem Res.

[98] Duntas LH. (2006). The Role of Selenium in Thyroid Autoimmunity and
Cancer. Thyroid.

[99] Fischer EG. (2020). Nuclear Morphology and the Biology of Cancer
Cells. Acta Cytol.

[100] Lacka K, Szeliga A. (2015). Significance of selenium in thyroid physiology and
pathology. Pol Merkur Lekarski.

[101] Shen F, Cai WS, Li JL, Feng Z, Cao J, Xu B. (2015). The Association Between Serum Levels of Selenium, Copper, and
Magnesium with Thyroid Cancer: A Meta-analysis. Biol Trace Elem Res.

[102] Jonklaas J, Danielsen M, Wang H. A (2013). Pilot Study of Serum Selenium, Vitamin D, and Thyrotropin
Concentrations in Patients with Thyroid Cancer. Thyroid.

[103] Kipp AP. (2020). Selenium in colorectal and differentiated thyroid
cancer. Hormones (Athens).

[104] Barrea L, Gallo M, Ruggeri RM, Giacinto PD, Sesti F, Prinzi N (2021). Nutritional status and follicular-derived thyroid cancer: An
update. Crit Rev Food Sci Nutr.

[105] Barchielli G, Capperucci A, Tanini D. (2022). The Role of Selenium in Pathologies: An Updated
Review. Antioxidants (Basel).

[106] de Oliveira Maia M, Batista BAM, Sousa MP, de Souza LM, Maia CSC. (2020). Selenium and thyroid cancer: a systematic review. Nutr Cancer.

